# Comprehensive Characterization of the Microbiological and Quality Attributes of Traditional Sicilian Canestrato Fresco Cheese

**DOI:** 10.3390/foods14173123

**Published:** 2025-09-06

**Authors:** Chiara Pisana, Margherita Caccamo, Marcella Barbera, Giovanni Marino, Graziella Serio, Elena Franciosi, Luca Settanni, Raimondo Gaglio, Cinzia Caggia

**Affiliations:** 1 Department of Agriculture, Food and Environment, University of Catania, Via S. Sofia, 100, 95123 Catania, Italy; chiara.pisana@phd.unict.it (C.P.); cinzia.caggia@unict.it (C.C.); 2 Consorzio per la Ricerca nel Settore della Filiera Lattiero-Casearia e dell’Agroalimentare (CoRFiLaC), 97100 Ragusa, Italy; caccamo@corfilac.it (M.C.); g.marino@corfilac.it (G.M.); 3 Department of Earth and Marine Sciences, University of Palermo, Via Archirafi, 90123 Palermo, Italy; marcella.barbera@unipa.it; 4 Department of Biological, Chemical and Pharmaceutical Sciences and Technologies, University of Palermo, Viale delle Scienze, 90128 Palermo, Italy; graziella.serio01@unipa.it; 5 Research and Innovation Centre, Fondazione Edmund Mach (FEM), Via E. Mach 1, 38098 San Michele all’Adige, Italy; elena.franciosi@fmach.it; 6 Department of Agricultural, Food and Forestry Science, University of Palermo, Viale delle Scienze 4, 90128 Palermo, Italy; luca.settanni@unipa.it

**Keywords:** traditional cheese, raw cow’s milk cheese, microbiological traits, next-generation sequencing, chemical characteristics, sensory test

## Abstract

This study provides a comprehensive characterization of the microbiological, chemical, and sensory profiles of Sicilian Canestrato Fresco (SCF) cheese, a traditional agri-food product (TAP) made from raw cow’s milk using artisanal methods and typically consumed after 20 d of ripening. Plate count analyses confirmed high levels of mesophilic lactic acid bacteria (LAB) exceeding 10^8^ CFU/g. Both rod- and coccus-shaped LAB populations were present at these elevated levels. Pathogens such as *Listeria monocytogenes* and *Salmonella* spp. were not detected, although potential contaminants including *Enterobacteriaceae*, total coliforms, and *Escherichia coli* were detected at levels of 1.0–3.5 log CFU/g. High-throughput sequencing confirmed LAB as the dominant taxa, comprising the majority of the bacterial community, which accounted for 78.12% to 99.63% of the total relative abundance (RA) across all cheese samples. The fatty acid profile was typical of cow’s milk cheeses, with long-chain fatty acids (C15–C18) representing ~75% of the total, followed by medium- (~17%) and short-chain (<8%) fatty acids. Volatile organic compound analysis showed free fatty acids as the most abundant class, followed by esters, alcohols, ketones, and aldehydes. These findings highlight the role of traditional practices in preserving the sensory and chemical identity of SCF cheese. However, the presence of hygiene indicators suggests a need for improving sanitary measures along the production chain. Future research should explore the impact of targeted microbial management and packaging conditions to enhance both safety and product standardization without compromising artisanal traits.

## 1. Introduction

In recent years, traditional cheeses have garnered renewed global interest, driven by a growing appreciation for food heritage, biodiversity, and artisanal production practices [1]. This resurgence has led to a steady increase in consumer demand, with traditional dairy products gaining traction not only among niche markets but also within mainstream retail and food service sectors. These cheeses are valued for their cultural authenticity, unique sensory profiles, and strong market appeal [2]. Typically produced by small-scale, often family-run farms, traditional cheeses are made using raw milk from local animal breeds, traditional wooden tools, and without the use of commercial starter cultures [3]. As a result, their fermentation relies on the natural microbiota present in raw milk, animal-derived rennet, wooden processing equipment, and the surrounding cheese-making environment [4].

Sicily has a long-standing tradition of cheese-making; five Sicilian cheeses have been awarded Protected Designation of Origin (PDO) status [5], while another 25 are officially recognized as traditional agri-food products (TAPs) by the Italian Ministry of Agriculture, Food Sovereignty and Forestry [6]. Among these is Sicilian Canestrato Fresco (SCF), locally known as “Canistratu”, a historic cheese with documented origins dating back to the early 15th century. Historical documentation, including the “Calmiere dei latticini” of 1407 and the municipal price list of 1412, both preserved in the Palermo State and Municipal Archives, attests to the longstanding presence of this cheese in the regional dairy economy [7,8].

This cheese represents a cornerstone of Sicilian culinary tradition, widely appreciated by consumers for its delicate flavor and broad application in cuisine. Commercially classified as a semi-hard cheese, it is commonly enjoyed as an appetizer, either on its own or paired with complementary ingredients such as olive oil or honey [9].

**Figure 1:**
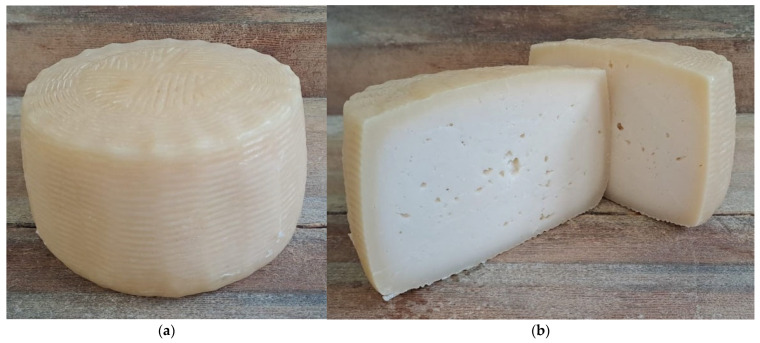
Sicilian Canestrato Fresco cheese. (**a**) Whole wheel; (**b**) cut section.

SCF cheese is produced by applying pressed cheese technology from raw cow’s milk or a mixture of raw cow, ewe, and goat milk. It is characterized by a typical cylindrical shape of 1–2 kg, a uniform texture, and an ivory-white color, and is marketed after a ripening period of 15–20 d [10] (Figure 1).

From a food safety perspective, the use of raw milk combined with short ripening periods in the production of SCF cheese requires greater attention compared to cheeses made from thermally treated milk, as the final products may retain pathogenic microorganisms from the raw milk that can survive the cheese-making process [11]. Moreover, like all short-ripened cheeses, SCF cheese possesses physicochemical characteristics such as high moisture content, high pH, and low NaCl content, which create a favorable environment for the growth and persistence of different microbial groups [12].

Despite its cultural and historical importance, SCF cheese remains scientifically underexplored. To date, no comprehensive study is available on its microbiological and physicochemical characteristics. This represents a significant knowledge gap, particularly in light of the increasing interest in traditional and raw milk cheeses. A deeper understanding of SCF’s microbial ecology and quality attributes appeared essential not only for ensuring product safety and consistency but also for supporting its valorization and protection within the artisanal dairy sector. This research presents the first in-depth investigation of the microbiological and chemical properties of SCF cheese produced from raw cow’s milk following traditional artisanal protocol.

This study is part of a research initiative aimed at characterizing lesser-known traditional Sicilian dairy products, preserving their heritage, and enhancing their visibility in both national and international markets. Specifically, the objectives of this study were to evaluate SCF cheeses for the (i) microbiological properties by culture-dependent and -independent approaches; (ii) proximate compositional attributes, antioxidant properties, and fatty acid profiles; (iii) volatile organic compounds; and (iv) sensory traits.

## 2. Materials and Methods

### 2.1. Sample Collection

**Figure 2:**
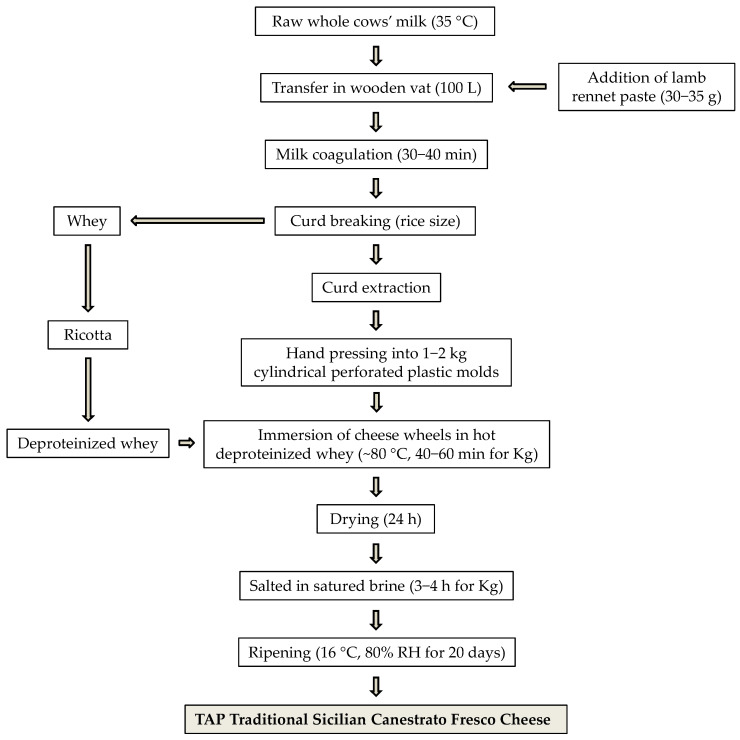
Flowchart of traditional agri-food products (TAPs): Sicilian Canestrato Fresco cheese production. Abbreviations: RH, relative humidity.

Samples of SCF cheese were collected from six dairy factories located in the province of Ragusa, in eastern Sicily, during the winter 2024 production period. The factories were selected based on their exclusive use of raw cow’s milk and strict adherence to the traditional manufacturing protocol defined by the Sicilian TAP production process [13] (Figure 2), which includes the use of wooden equipment and excludes the addition of starter cultures.

Moreover, all production factories utilize the same dairy cattle breed (Italian Holstein-Friesian) and follow grazing-based management practices, where the animals primarily graze on natural pasture. Cheese samples were collected after 20 d of ripening, with the process repeated in triplicate over three consecutive weeks. At each sampling interval, the cheeses were transported in a portable refrigerator to the Microbiology Laboratory of the Consorzio per la Ricerca nel Settore della Filiera Lattiero-casearia e dell’agroalimentare (CoRFiLaC, Ragusa, Italy) and to the Laboratory of Agricultural, Food and Environmental Microbiology (University of Palermo).

### 2.2. Culture-Dependent Microbiological Assessment of Cheeses

All collected samples of SCF cheese underwent microbiological analyses to assess the viable levels of the main microbial groups commonly associated with dairy products. For each analysis, 25 g of cheese was aseptically homogenized in 225 mL of a 2% (*w*/*v*) sodium citrate (Sigma-Aldrich, Milan, Italy) solution, using a stomacher (VWR International, Darmstadt, Germany). The homogenized samples were subjected to serial ten-fold dilutions (1:10) in Ringer’s solution (Oxoid, Hampshire, United Kingdom), after which the resulting cell suspensions were plated onto selective agar media. Specifically, mesophilic lactic acid bacteria (LAB) rods were enumerated on de Man–Rogosa–Sharpe (MRS; VWR International, Darmstadt, Germany) agar; mesophilic LAB cocci on Mediun 17 (M17; BioMaxima, Lublin, Poland) agar, incubated at 30 °C for 2 d; enterococci on Kanamycin Esculin Azide (KAA; VWR International, Darmstadt, Germany) agar, incubated at 37 °C for 1 d; *Pseudomonadaceae* on *Pseudomonas* Agar Base (PAB; VWR International, Darmstadt, Germany), incubated at 25 °C for 2 d; members of the *Enterobacteriaceae* family on Violet Red Bile Glucose Agar (VRBGA; Neogen, Lansing, MI, USA), incubated at 37 °C for 1 d; total coliforms on Violet Red Bile Agar (VRBA; Lickson, Vicari, Italy), incubated at 37 °C for 1 d; *Escherichia coli* on Tryptone Bile Agar (TBX; VWR International, Darmstadt, Germany) agar, incubated at 37 °C for 1 d; coagulase-positive staphylococci (CPS) on rabbit plasma fibrinogen (VWR International, Darmstadt, Germany) agar, incubated at 37 °C for 1 d; yeasts and molds on Oxytetracycline Glucose Yeast Extract (OGYE; Liofilchem, Roseto degli Abruzzi, Italy) agar, incubated at 28 °C for 2 d and 25 °C for 5 d, respectively. In addition, the detection of *Salmonella* spp. and *Listeria monocytogenes* was performed following the standardized procedures outlined in ISO 6579-1 [14] and ISO 11290-1 [15], respectively. All plate counts were incubated under aerobic conditions, except for those for the enumeration of LAB, which were incubated anaerobically in hermetically sealed jars using AnaeroGen™ L 2.5 atmosphere generation systems (Thermo Fisher Scientific, Waltham, MA, USA). All microbiological counts were carried out in triplicate for all samples at each sampling time.

### 2.3. Culture-Independent Profiling of the Bacterial Community of Cheeses

Genomic DNA was extracted from SCF cheese samples using the DNeasy PowerFood Microbial Kit (QIAGEN, Hilden, Germany), following the protocol provided by the manufacturer to maximize extraction efficiency and DNA integrity. The quality of the extracted DNA was evaluated through agarose gel electrophoresis to assess integrity, and by UV/Vis spectrophotometric analysis to determine purity and concentration. As described by Busetta et al. [16], amplification of the V3-V4 hypervariable regions of the bacterial 16S rRNA gene was carried out using the primer pair 341F (5′-CCTACGGGNGGCWGCAG-3′) and 806R (5′-GACTACNVGGGTWTCTAATCC-3′). Following PCR amplification, the resulting libraries underwent purification using AMPure XP magnetic beads (Beckman Coulter, Brea, CA, USA) and were subsequently subjected to high-throughput sequencing on the Illumina^®^ MiSeq platform (Illumina Inc., San Diego, CA, USA). Raw sequence data were processed using the DADA2 pipeline [17], which included quality filtering, trimming of low-quality regions, error correction (denoising), and merging of paired-end reads. Taxonomic classification of bacterial sequences was conducted using the Greengenes database (version 13_8, 99%) to assign Operational Taxonomic Units (OTUs). The resulting FASTQ files were submitted to the NCBI Sequence Read Archive (SRA) under the accession number PRJNA1298803.

### 2.4. Proximate Analysis and Antioxidant Properties of Cheeses

Proximate compositional attributes of SCF cheese samples, including moisture, fat, protein, and NaCl content, were quantitatively determined using the FoodScan™ 2 analyzer (Foss Electric A/S, Hillerød, Denmark), based on mid-infrared spectroscopy (MIRS) prediction models, following the methodology described by Scatassa et al. [18].

To assess the total polyphenolic content (TPC) and antioxidant activity, three independent cheese samples (2 g each) were extracted using 70% (*v*/*v*) aqueous ethanol, applying a solvent-to-sample ratio of 3:1 (mL/g). The mixtures were subjected to ultrasonic treatment for 10 min to enhance cell disruption and facilitate the release of extractable compounds. This was followed by 24 h of maceration at room temperature, with intermittent agitation to ensure thorough extraction. After extraction, the samples were centrifuged at 10,000× *g* for 10 min at 4 °C, and the resulting supernatants were filtered through 0.45 μm membrane filters (Millex-HV, Millipore Corporation, Billerica, MA, USA) to obtain clear extracts. These were subsequently stored at −20 °C until further analysis.

The TPC was quantified using the Folin–Ciocalteu colorimetric assay, following the protocol described by Singleton et al. [19]. This method relies on the reduction of the Folin–Ciocalteu reagent by phenolic hydroxyl groups under alkaline conditions, forming a blue chromophore measurable at 735 nm. A gallic acid standard curve was used for calibration, and results were expressed as mg gallic acid equivalents (GAE) per 100 g of fresh weight (FW). Antioxidant activity was evaluated using the ferric-reducing antioxidant power (FRAP) assay, as outlined by Mannino et al. [20]. The assay employed a freshly prepared reagent composed of 300 mM sodium acetate trihydrate solution in glacial acetic acid (pH 3.6) (Sigma Aldrich, St. Louis, MO, USA), 10 mM TPTZ in HCl (Sigma Aldrich, St. Louis, MO, USA), in HCl (Biosolve B.V., Valkenswaard, Netherlands) and 20 mM ferric chloride hexahydrate (Sigma Aldrich, St. Louis, MO, USA), mixed in an 8:1:1 ratio. Diluted extracts were incubated with the FRAP reagent at 37 °C for 30 min, promoting the reduction of Fe^3+^–TPTZ to Fe^2+^–TPTZ. The increase in absorbance at 600 nm was measured spectrophotometrically, and antioxidant capacity was expressed as µmol Trolox equivalents (TE) per 100 g of FW. All measurements were conducted in triplicate to ensure analytical reproducibility and reliability.

### 2.5. Determination of Cheese Fatty Acids

The fatty acid profile of the cheese samples was determined using gas chromatography–mass spectrometry (GC–MS; Agilent Technologies, Santa Clara, CA, USA). Lipid extraction was carried out according to the method described by De Jong and Badings [21]. After extraction, fatty acids were converted to fatty acid methyl esters (FAMEs) by methylation with 2 mL of 0.5 M NaOCH_3_ at 50 °C for 15 min, followed by 1 mL of 5% HCl in methanol at 50 °C for 15 min [22]. An aliquot of 1 μL from the extracted fraction was analyzed using the GC–MS system with a split injection ratio of 1:40. Chromatographic separation was performed on a DB-WAX capillary column (60 m × 0.25 mm i.d. × 0.25 μm film thickness), using helium as the carrier gas at a constant flow rate of 1 mL/min. The oven temperature program started with an initial hold at 50 °C for 1 min, followed by a ramp to 200 °C at 25 °C/min with a 10 min hold, and then increased to 230 °C at 3 °C/min, held for 26 min. The injector and detector temperatures were set at 250 °C and 300 °C, respectively. Identification and quantification of individual fatty acids were performed by comparing retention times with those of a certified reference mixture (Supelco 37 Component FAME Mix, Sigma-Aldrich, St. Louis, MO, USA). Each sample was analyzed in triplicate.

### 2.6. Volatile Organic Compound Analysis

Volatile organic compounds (VOCs) of SCF cheese samples were extracted using a headspace solid-phase microextraction (SPME) fiber (DVB/CAR/PDMS, 50 mm, Supelco, Bellefonte, PA, USA). The VOCs were analyzed with a gas chromatograph (Agilent 6890, Agilent Technologies, Santa Clara, CA, USA) coupled to a mass selective detector (Agilent 5975C, Agilent Technologies, Santa Clara, CA, USA) and fitted with a DB-624 capillary column (Agilent Technologies, 60 m × 0.25 mm × 1.40 µm, Santa Clara, CA, USA). For the analysis, 5 g of cheese was finely chopped and placed in a 25 mL glass vial. The sample was then exposed to the SPME fiber at 60 °C for 15 min under constant stirring. Thermal desorption of the VOCs from the fiber was carried out in a splitless injector at 250 °C for 1 min. Chromatographic separation was performed using helium as the carrier gas at a flow rate of 1 mL/min. The oven temperature was initially set at 40 °C for 5 min, then increased at a rate of 5 °C/min until reaching 200 °C, where it was maintained for an additional 2 min. VOCs were detected in full scan mode using mass spectrometry, with an interface temperature of 230 °C and a mass range of 40–400 m/z. Compound identification was based on comparison of the obtained mass spectra with those in the NIST library. The relative abundance of each compound was calculated by normalizing the peak area to the total area of all significant peaks. Each sample was analyzed in triplicate.

### 2.7. Sensory Analysis

The Check-All-That-Apply (CATA) method was employed to gather consumer perceptions of SCF cheese samples. Based on chemical analyses and the nature of the CATA test, a pooled set of SCF sample, prepared by combining equal amounts of cheese from each dairy factor, was presented to consumers for visual evaluation. Cheese portions (approximately 10 g) were cut into uniform slices and equilibrated to room temperature (20 ± 2 °C) for 30 min prior to sensory analysis. Each participant evaluated one sample of SCF cheese, identified by a three-digit code and served on odorless, disposable trays. All participants were regular cheese consumers (consuming cheese at least once per week) and were recruited from among customers of a local store. Informed consent was obtained prior to participation.

The CATA questionnaire included 13 sensory descriptors, developed through a preliminary focus group of expert panelists (*n* = 4) and refined via expert review, following methodologies described by Ares and Jaeger [23]. The list encompassed terms related to appearance (white color, yellow color, presence of holes, presence of cracks, compactness), odor (milky, whey, typical), and texture (crumbly, gummy, soft, hard). Consumers were instructed to visually analyze, smell, and touch each sample, and to check all attributes they considered appropriate. No training or reference standards were provided, in accordance with the consumer-centered nature of the method [24,25].

### 2.8. Statistical Analyses

Microbial counts of SCF cheese samples were log-transformed to normalize data distribution and were graphically represented using box-and-whisker plots. A one-way ANOVA was conducted on the chemical data, followed by Tukey’s post hoc test for pairwise comparisons. These analyses were performed using XLStat software, version 2020.3.1 for Excel (Addinsoft, New York, NY, USA). The frequency of mention for each CATA descriptor was calculated for each cheese sample. Statistical significance was set at *p* < 0.05.

## 3. Results and Discussion

### 3.1. Microbiological Profile of Cheeses

**Figure 3:**
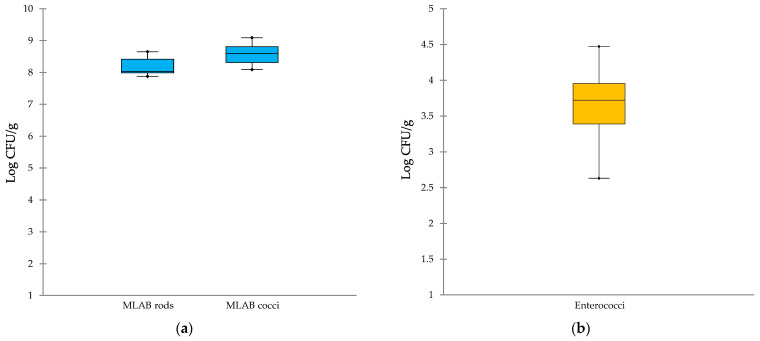

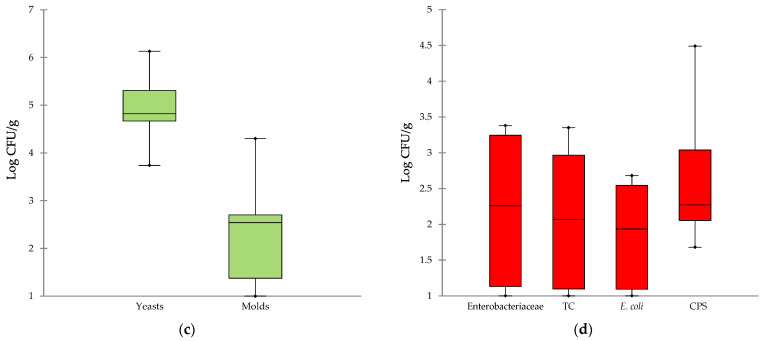
Box-and-whisker plots of microbial loads of Sicilian Canestrato Fresco cheeses. (**a**) Lactic acid bacteria; (**b**) enterococci; (**c**) spoilage microorganisms; (**d**) potentially pathogenic bacteria. Abbreviations: MLAB, mesophilic lactic acid bacteria; TC, total coliforms; *E*., *Escherichia*; CPS, coagulase-positive staphylococci.

The microbiological profile of SCF cheese samples was assessed through plate count analyses targeting key microbial groups commonly associated with dairy ecosystems. These included beneficial pro-technological bacteria as well as spoilage and potential pathogenic microorganisms (Figure 3).

A focused microbiological assessment was conducted to detect members of the *Pseudomonadaceae* family, known for their spoilage potential in short-ripened cheeses due to proteolytic and lipolytic activities that compromise texture and induce off-flavors [26], as well as *Salmonella* spp. and *L. monocytogenes*, which are major contributors to global foodborne outbreaks [27]. None of these microorganisms were detected in any of the SCF cheese samples, and therefore, they are not represented in Figure 3.

Mesophilic LAB, both rod- and coccus-shaped, were consistently found at cell densities exceeding 10^8^ CFU/g, with minimal variability among samples (Figure 3a). The scalding phase applied post-pressing, involving immersion in hot deproteinized whey for approximately one hour, was not effective in significantly reducing thermoduric LAB. This is primarily due to the limited heat penetration into the cheese mass, as internal temperatures remain substantially lower than the ~80 °C temperature of the whey. Indeed, Todaro et al. [28] investigated this phenomenon in Pecorino cheeses using Thermo Button data loggers placed at molding in both the core and under-rind sections. They observed that the internal temperature at the end of the immersion phase did not exceed 43.6 °C. The strong and stable presence underscores the dominant role of LAB in the cheese microbiota and their essential contribution to acidification, proteolysis, and flavor development during early ripening stages [29]. These bacteria are typically derived from raw milk and the dairy environment [4], and their persistence throughout the production process reflects the selective pressure exerted by the cheese matrix, favoring strains well-adapted to the artisanal conditions of SCF production.

Enterococci (Figure 3b), a group with debated implications for human health due to their role as beneficial, opportunistic, or undesired organisms [30,31,32], were present at cell densities ranging from 10^3^ to 10^5^ CFU/g, values commonly found in fresh cheeses made from raw cow’s or ewe’s milk [33,34].

Yeasts and molds, known for their capacity to compromise the quality and shelf life of short-ripened cheeses, were detected at variable levels across SCF cheese samples (Figure 3c), with the highest value reaching 6.13 and 4.30 log CFU/g, respectively. These microorganisms can colonize cheese surfaces and promote unwanted fermentative processes, leading to visible defects, textural degradation, and the development of off-flavors [35]. Their metabolic activity may also result in gas formation, discoloration, and the production of undesirable volatile compounds, particularly in cheeses with high moisture content and limited ripening time [36]. The observed variability in yeast and mold counts likely reflects differences in environmental hygiene and storage conditions during and after cheese production. In artisanal settings, where exposure to open environments and manual handling are common, contamination from air, equipment, or packaging materials is a frequent occurrence [37,38]. Therefore, implementing improved sanitation protocols is essential to limit fungal contamination.

Potentially pathogenic bacteria, such as *Enterobacteriaceae*, total coliforms, and *E. coli* showed consistent distribution across samples (Figure 3d), while CPS were detected at comparatively higher levels. These findings suggest that fecal contamination and poor hygienic conditions during processing are contributing factors [39]. Specifically, this contamination is primarily attributed to the transfer of these bacteria from the surface of the udder to the milk during milking procedures [39]. Overall, the data emphasize the importance of reinforcing hygiene and safety protocols throughout the SCF cheese production continuum, from milking to final packaging, to safeguard product quality and consumer health.

### 3.2. Composition of Cheese Bacterial Communities

In the present study, high-throughput sequencing using Illumina technology was employed to investigate the bacterial communities present in SCF cheese samples. This culture-independent metagenomic approach offers a powerful tool for profiling microbial ecosystems in complex food matrices, enabling the identification of both cultivable and non-cultivable taxa [40]. Unlike traditional culturing techniques, which are limited to detecting metabolically active and fast-growing microorganisms, sequencing-based methods can also uncover dormant or slow-growing bacterial populations, as well as those that may be metabolically inactive at the time of sampling [41]. This comprehensive insight is particularly valuable in fermented foods, where microbial diversity plays a crucial role in shaping sensory attributes and safety aspects.

**Figure 4:**
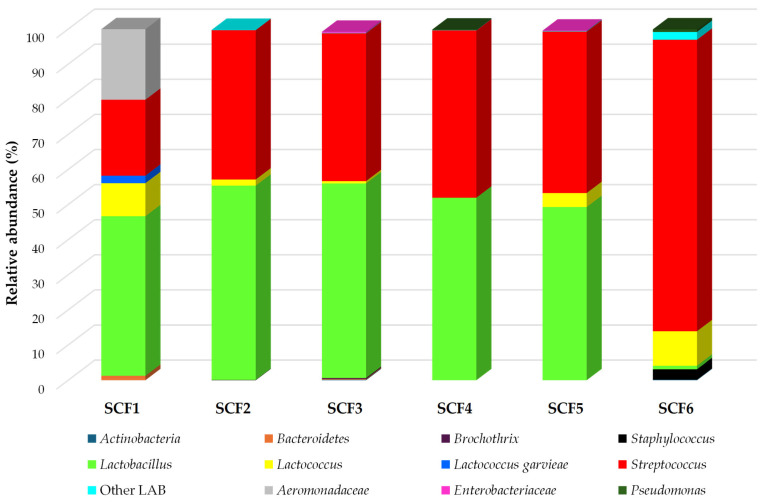
Relative abundances (%) of bacterial community identified in Sicilian Canestrato Fresco cheeses. Abbreviations: SCF, Sicilian Canestrato Fresco cheese; 1–6, dairy factory.

As illustrated in Figure 4, the analysis focused on operational taxonomic units (OTUs) with a relative abundance (RA) ≥ 0.1%, which are considered representative of the dominant microbiota [42].

A total of twelve distinct taxonomic groups were identified, primarily at the genus level, reflecting the microbial complexity of SCF cheeses (Table S1). Among these, LAB, notably *Lactobacillus*, *Lactococcus*, and *Streptococcus*, were consistently detected across all samples, with cumulative RA ranging from 78.12% to 99.63%. These genera are well-known for their pivotal roles in cheese fermentation and ripening, contributing to acidification, proteolysis, and flavor development [43].

*Streptococcus* emerged as the most abundant genus, with RA values spanning from 21.65% in SCF1 to 83.06% in SCF6, followed by *Lactobacillus*, which showed a broader distribution (1.00% in SCF6 and 55.37% in SCF3). The presence of both starter and non-starter LAB within these genera underscores their functional importance throughout the cheese production process. These findings align with previous studies reporting similar microbial profiles in raw milk cheeses, where LAB dominate due to their adaptability to the cheese environment and their metabolic versatility [44].

Importantly, no sequences corresponding to *Salmonella* spp. or *L. monocytogenes* were detected in any of the samples, corroborating the results obtained through culture-based plate counting and confirming the microbiological safety trait of the analyzed cheese samples. However, a notable discrepancy was observed for members of the *Enterobacteriaceae* family and *Staphylococcus*, which, although at low RA (<1%) in the sequencing data, were consistently counted via culturing. This divergence may be attributed to DNA degradation caused by nuclease activity, which can compromise the integrity of bacterial DNA and hinder its amplification during sequencing [45]. Overall, the integration of high-throughput sequencing into the analysis of SCF cheeses provides a robust understanding of their microbial composition, offering useful insights for quality control and preservation of traditional cheese-making practices.

### 3.3. Proximate Composition and Antioxidant Properties of Cheeses

Table 1 presents the gross chemical composition of the SCF cheese samples, as determined using the FOSS analytical system. This method has been extensively employed in previous studies to accurately assess the chemical characteristics of dairy products [46,47], offering a reliable and standardized approach for compositional analysis. In the SCF samples, moisture content ranged from 34.81% to 37.52%, protein content from 25.56% to 28.37%, and fat content from 27.42% to 30.82%. These values fall within the expected range for fresh cheeses and are consistent with those reported by Gobbetti et al. [48] for traditional Italian cheeses produced from raw cow’s milk. Such alignment reinforces the representativeness of our samples and supports the validity of the analytical approach adopted.

The antioxidant properties of SCF cheese were robustly demonstrated through the combined evaluation of TPC and FRAP (Table 1). The TPC values, which ranged from 120 to 140 mg gallic acid equivalents (GAE) per 100 g of fresh weight (FW), indicate that SCF cheese can be considered a valuable dietary source of phenolic compounds. These bioactive molecules are primarily derived from the animals’ pasture-based diet, which is rich in polyphenol-containing plants and may be concentrated or bio-transformed during the early stages of microbial fermentation and cheese ripening. Polyphenols are widely recognized for their antioxidant, anti-inflammatory, and antimicrobial properties, and their presence in dairy products has been associated with enhanced nutritional and functional value [49]. In alignment with the TPC data, the FRAP assay revealed a moderate but significant reducing capacity, with values ranging from 18.71 to 21.51 µmol TE per 100 g of FW. This confirms that the phenolic fraction contributes substantially to the overall antioxidant potential of SCF cheese. The positive correlation between TPC and FRAP values suggests that the antioxidant activity is largely attributable to phenolic hydroxyl groups, which are known to effectively reduce metal ions and inhibit oxidative reactions [50]. The retention of these bioactive compounds within the cheese matrix may offer dual benefits: enhancing the nutritional profile of the product and contributing to its oxidative stability. Antioxidants, such as polyphenols, can delay lipid peroxidation, thereby preventing the formation of off-flavors and extending the shelf life of the cheese [51]. These functional attributes support consumer health and improve product quality during storage and distribution.

Furthermore, the presence of naturally occurring antioxidants in SCF cheese underscores the importance of traditional production methods and local agri-food heritage. By preserving pasture-based feeding systems and artisanal cheese-making practices, SCF emerges as a product that combines high nutritional value with distinctive sensory characteristics, aligning with contemporary consumer preferences for functional and sustainable foods.

### 3.4. Fatty Acid Composition of Cheeses

Table 2 reports the FA profiles of the six SCF cheese samples, expressed as percentages of total FA. The data reveal a consistent distribution pattern across all samples, characterized by a predominance of LCFA (C15–C18), followed by MCFA (C10–C14) and SCFA (C6–C10).

The abundances were approximately 75% for LCFA, 17% for MCFA, and less than 8% for SCFA. This hierarchical distribution is in line with well-established profiles of cow dairy products, where LCFAs dominate due to the biosynthetic activity of the mammary gland and the metabolic processes occurring in the rumen [52].

Among the LCFAs, saturated fatty acids (SFAs) were the most prevalent, with palmitic acid (C16:0) consistently emerging as the dominant component across all samples, followed by myristic acid (C14:0) and stearic acid (C18:0), both of which are typical of cow milk fat. Within the monounsaturated fatty acid (MUFA) fraction, oleic acid (C18:1 cis9) was the most abundant. These findings are consistent with previous studies on similar cheese productions [53,54], reinforcing the notion that the FA profile of SCF cheeses reflects typical bovine milk fat composition [55]. Importantly, FA profiles did not exhibit significant variation among the different cheese batches, suggesting a high degree of consistency in the production process. This uniformity likely stems from standardized protocols and controlled processing conditions shared across the production sites [52,56]. It is worth noting that while FA composition can be influenced by a range of factors, including animal species, dietary regimen, cheese-making technology, and ripening duration, variations are generally minimal when cheeses are produced under similar conditions [57,58,59,60,61,62]. Therefore, the observed homogeneity in FA profiles across samples further supports the reproducibility and reliability of the SCF cheese production system.

### 3.5. Volatilome Composition

**Figure 5:**
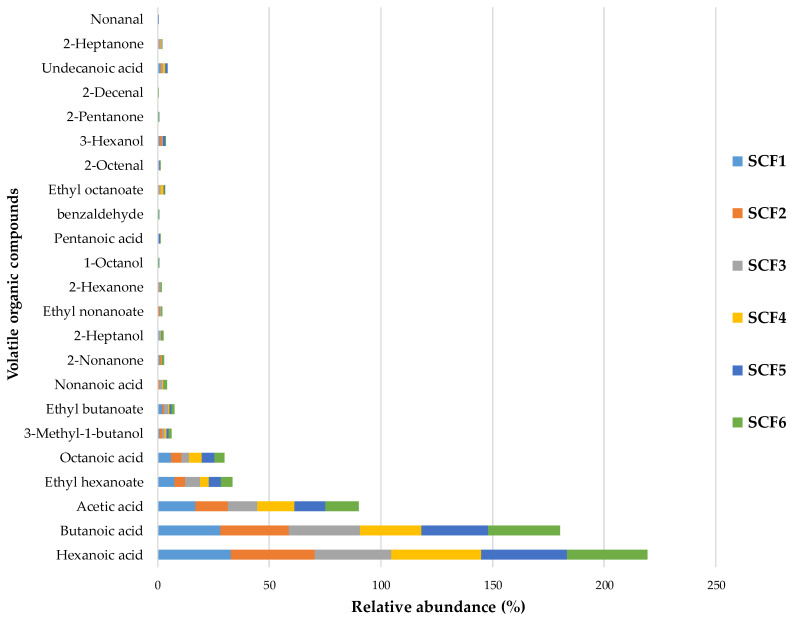
Distribution of volatile organic compounds in cheese samples. Results are reported as percentage of triplicate determinations conducted at each sampling time point (peak area of each compound/total area of significant peaks) ×100. Abbreviations: SCF, Sicilian Canestrato Fresco cheese; 1–6, dairy factory.

The VOC profiles emitted from SCF cheese samples are reported in Figure 5. The analysis revealed a consistent pattern across all samples, with free fatty acids (FFAs) emerging as the most dominant VOC group (85.3–91.8%), followed by esters (5.7–10.7%), alcohols (1.3–2.7%), ketones (0.5–1.7%), and aldehydes (0.1–1.2%) (Table S2).

This predominance of FFAs is characteristic of cow’s milk cheeses and reflects the primary lipolytic activity occurring during cheese maturation [63,64]. Lipolysis in these cheeses is largely driven by the enzymatic activity of native lipoprotein lipase and is further enhanced by the use of traditional lamb rennet during curd formation [65,66]. Within this group, hexanoic and butanoic acids were consistently the most abundant across all samples. These compounds are known contributors to the sharp, pungent, and sometimes rancid notes typical of aged cheeses, and their presence aligns with findings from similar studies on traditional dairy products [59,60].

Esters represented the second most abundant VOC class. These compounds are formed through the esterification of fatty acids and alcohols, and they play a crucial role in modulating the sensory profile of cheese by introducing fruity and floral notes that balance the sharpness of FFAs. In particular, ethyl hexanoate and ethyl butanoate were the most prominent esters detected, both of which are associated with pleasant aromatic traits and are known to reduce bitterness derived from amines [67,68,69]. Similar ester profiles have been documented in traditional Sicilian cheeses such as Provola dei Nebrodi [70], suggesting a regional consistency in aromatic traits.

Although present in lower concentrations, alcohols, aldehydes, and ketones contributed significantly to the overall aromatic complexity. Notably, 3-methyl-1-butanol, a branched-chain alcohol, imparted herbaceous and malty notes, enhancing the depth of the aroma profile [65,71,72].

Aldehydes, such as nonanal, 2-decenal, and benzaldehyde were associated with green grass, almond-like, and floral aromas, adding subtle nuances to the sensory experience [72,73,74]. Ketones, including 2-heptanone, 2-nonanone, and 2-pentanone, contributed cheesy, fruity, and citrus-like notes, respectively, further enriching the flavor profile [65,71,75].

Overall, the VOC composition was remarkably homogeneous across all SCF cheese samples. This uniformity likely reflects the use of standardized artisanal manufacturing practices, including consistent milk sourcing, rennet application, and curd-handling techniques [76]. These findings are in line with previous research on Canestrato di Moliterno, where Faccia et al. [53] observed that significant sensory differences among producers emerged only when ripening conditions varied substantially. Similarly, Trani et al. [77] reported that cheeses produced under a uniform production protocol exhibited homogeneous sensory and volatile profiles, underscoring the importance of controlled processing in maintaining product consistency.

The present data confirm the typical VOC fingerprint of traditional cow’s milk cheeses and highlight the delicate balance among enzymatic activity, microbial metabolism, and artisanal practices in shaping the sensory identity of SCF cheeses.

### 3.6. Sensory Evaluation

**Figure 6:**
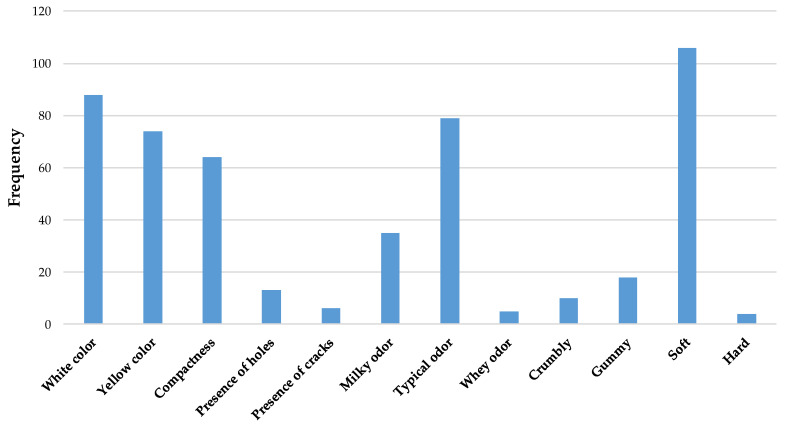
Frequency distribution of sensory attributes, expressed as absolute values, identified by consumers (*n* = 179) during Check-All-That-Apply (CATA) test of Sicilian Canestrato Fresco cheese.

The results of the CATA test conducted on SCF cheese samples provided valuable insights into consumer-perceived sensory attributes. A total of 179 consumers (85 females, 94 males) participated in the study, contributing to a robust sample size for sensory profiling. The most frequently selected descriptors included “white color,” “yellow color,” “compactness,” “typical odor,” and “softness” (Figure 6). These results suggest that visual appearance, texture, and aromatic identity are key drivers in consumer perception and characterization of SCF cheese. The simultaneous high frequency of both “white” and “yellow” color descriptors may reflect natural variability in the surface and interior coloration of the cheese, which is common in artisanal products made from raw milk and without standardized coloring agents. This dual citation pattern also highlights that color perception may vary among consumers, potentially influenced by lighting conditions, expectations, or sample presentation [23]. The selection of “compactness” and “softness” as dominant texture-related attributes may initially appear contradictory, but they can coexist in freshly pressed cheeses. In its short-ripened state, Canestrato Fresco often presents a firm but yielding body, which consumers may perceive as both dense and soft depending on applied pressure. Despite its status, SCF was frequently described as compact, indicating a firm but elastic texture typical of pressed short-ripened cheeses. This is consistent with traditional processing practices, where curd is pressed and briefly aged to give the cheese a defined, sliceable body [78]. This dual perception also aligns with findings from previous research on fresh and semi-hard cheeses, where consumers reported overlapping texture categories [25]. The frequent mention of “typical odor” underscores the importance of olfactory cues in identifying authenticity and typicality in traditional cheeses. The term likely reflects consumer familiarity with the characteristic aromatic profile of Canestrato Fresco, which includes lactic, grassy, or mildly animal notes resulting from raw milk and artisanal production methods.

Similar observations have been reported in CATA studies on other traditional cheeses, where descriptors such as “typical” or “authentic” odor were frequently associated with a positive product identity [21]. The descriptor “milky odor” was selected with moderate frequency, suggesting that while the cheese retains some fresh lactic notes, these are not the dominant aromatic feature for most consumers. Several factors may account for this outcome, including the presence of more complex aromas derived from raw milk and traditional processing methods, which may mask or overshadow the simpler “milky” character. Additionally, lexical overlap between “milky odor” and broader terms like “typical odor” may have led some participants to favor the latter when describing the cheese [21].

## 4. Conclusions

SCF is a traditional short-ripened pressed cheese made from raw cow’s milk, typically marketed after a short ripening period of approximately 20 days. While this limited maturation enhances its fresh sensory profile and preserves its artisanal character, it may also pose microbiological risks, particularly concerning the potential presence of foodborne pathogens. This study demonstrated that applying traditional production protocols during the winter season promotes the establishment and dominance of LAB typically associated with dairy environments. These microbial communities play a key role in shaping the biochemical and sensory properties of the cheese, although their composition may vary seasonally. However, the detection of fecal contaminants underscores the need for further investigation into hygiene practices and microbial safety throughout the production chain. Future research should focus on the functional characterization of dominant LAB and enterococcal strains collected across all four seasons. Such insights would be essential for balancing the preservation of traditional methods with modern food safety standards. From a compositional perspective, the proximate chemical parameters, FA profiles, and VOC patterns were remarkably consistent across the different dairy factories involved in this study. This homogeneity suggests that strict adherence to shared artisanal protocols ensures a high degree of reproducibility in the final product, despite potential variability in raw milk or environmental conditions. VOC analysis revealed a stable aromatic profile dominated by FFAs and esters, with minor but meaningful contributions from alcohols, ketones, and aldehydes. These findings highlight the importance of microbial and enzymatic activity in flavor development, reinforcing the role of traditional practices in maintaining the sensory identity of SCF cheese.

In conclusion, while enhancing hygiene and safety measures across all stages of SCF cheese production is essential, preserving and carefully applying traditional protocols remains the most effective strategy for ensuring product consistency, quality, and cultural authenticity. A deeper understanding of the microbial ecology and biochemical dynamics of SCF will be crucial for supporting its valorization and safeguarding its future in both local and broader markets.

## Figures and Tables

**Table 1:** Proximate composition of Sicilian Canestrato Fresco cheeses.

Parameters	Samples						SEM	*p*-Value
	SCF1	SCF2	SCF3	SCF4	SCF5	SCF6		
Moisture (%)	36.11	36.55	35.42	36.48	34.81	37.52	0.27	0.707
Protein (%)	28.37	25.81	27.63	26.78	26.02	25.56	0.28	0.572
Fat (%)	28.71	27.42	27.30	26.38	30.82	27.87	0.30	0.187
NaCl (%)	2.03	1.53	1.58	1.74	1.73	1.91	0.04	0.153
TPC (mg GAE/100 g FW)	130.06	130.45	126.41	130.84	140.04	132.87	0.84	0.125
FRAP (µM TE/100 g FW)	18.71	21.51	19.14	21.16	20.02	18.99	0.20	0.057

Results represent the mean values obtained from triplicate determinations conducted at each sampling time point. Abbreviations: SCF, Sicilian Canestrato Fresco cheese; 1–6, dairy factory; SEM, standard error of mean; TPC, total polyphenol content; GAE, gallic acid equivalents; FRAP, ferric-reducing antioxidant power; TE, Trolox equivalent.

**Table 2:** Fatty acid profile of Sicilian Canestrato Fresco cheeses.

Fatty Acids (g/100 g FA)	Samples						SEM	*p*-Value
	SCF1	SCF2	SCF3	SCF4	SCF5	SCF6		
Caproic acid (C6:0)	2.41	2.40	2.46	2.44	2.39	2.48	0.02	0.781
Caprylic acid (C8:0)	1.61	1.72	1.70	1.74	1.69	1.63	0.01	0.083
Capric acid (C10:0)	3.75	3.71	3.85	3.72	3.80	3.79	0.02	0.622
Lauric acid (C12:0)	4.38	4.49	4.40	4.50	4.39	4.46	0.02	0.855
Myristic acid (C14:0)	12.90	12.85	12.95	12.80	12.83	13.00	0.06	0.995
Pentadecanoic acid (C15:0)	1.35	1.40	1.36	1.39	1.38	1.37	0.01	0.889
Palmitic acid (C16:0)	34.26	34.39	34.19	34.80	34.44	34.66	0.15	0.990
Palmitoleic acid (C16:1)	1.95	2.00	1.92	1.98	1.96	2.01	0.01	0.771
Stearic acid (C18:0)	11.77	11.82	11.77	11.68	11.80	11.76	0.04	0.858
Oleic acid [C18:1 (cis)]	20.65	20.70	21.02	20.40	20.55	20.45	0.10	0.950
Oleic acid [C18:1 (trans)]	1.55	1.50	1.46	1.4	1.42	1.49	0.01	0.196
Linoleic acid (C18:2)	2.92	2.91	2.89	2.86	2.90	2.85	0.01	0.972
∑SCFA	7.91	7.83	8.01	7.90	7.88	7.96	n.e.	n.e.
∑MCFA	17.31	17.34	17.48	17.39	17.20	17.56	n.e.	n.e.
∑LCFA	74.46	74.72	74.61	74.58	74.45	74.59	n.e.	n.e.

Results represent the mean values obtained from triplicate determinations conducted at each sampling time point. Abbreviations: SCF, Sicilian Canestrato Fresco cheese; 1–6, dairy factory; SEM, standard error of mean; n.e., not evaluated.

## Data Availability

The data that support the finding of this study are available from the corresponding author upon reasonable request.

## Supplementary Materials

The following supporting information can be downloaded at: https://www.mdpi.com/article/10.3390/foods14173123/s1, Table S1: Taxa composition revealed by high-throughput sequencing analysis in Sicilian Canestrato Fresco cheeses; Table S2: Volatile organic compounds determined by GC–MS in Sicilian Canestrato Fresco cheeses.
