# Supplementary material for: Comprehensive Characterization of the Microbiological and Quality Attributes of Traditional Sicilian Canestrato Fresco Cheese
Source: Foods. 2025 Sep 6;14(17):3123. doi: 10.3390/foods14173123 (PMC12427870; doi:10.3390/foods14173123)
Supplement: Supplementary file 1 [file foods-14-03123-s001.zip › foods-3834110-supplementary.pdf]

foods

MDPI

Article

Comprehensive Characterization of the Microbiological and Quality Attributes of Traditional Sicilian Canestrato Fresco Cheese

Chiara Pisana [1], Margherita Caccamo [2], Marcella Barbera [3], Giovanni Marino [2], Graziella Serio [4], Elena Franciosi [5], Luca Settanni [6], Raimondo Gaglio [6,*] and Cinzia Caggia [1,2]

[1] Department of Agriculture, Food and Environment, University of Catania, Via S. Sofia, 100, Catania, 95123, Italy; chiara.pisana@phd.unict.it (C.P.); cinzia.caggia@unict.it (C.C.)
[2] Consorzio per la Ricerca nel settore della Filiera Lattiero-Casearia e dell'agroalimentare (CoRFiLaC), 97100, Ragusa, Italy; caccamo@corfilac.it (M.C.); g.marino@corfilac.it (G.M.)
[3] Department of Earth and Marine Sciences, University of Palermo, Via Archirafi, Palermo, 90123, Italy; marcella.barbera@unipa.it (M.B.)
[4] Department of Biological, Chemical and Pharmaceutical Sciences and Technologies, University of Palermo, Viale delle Scienze, 90128 Palermo, Italy; graziella.serio@unipa.it (G.S.)
[5] Research and Innovation Centre, Fondazione Edmund Mach (FEM), Via E. Mach 1, 38098, San Michele all'Adige, Italy; elena.franciosi@fmach.it (E.F.)
[6] Department of Agricultural, Food and Forestry Science, University of Palermo, Viale delle Scienze 4, 90128 Palermo, Italy; luca.settanni@unipa.it (L.S.)
* Correspondence: raimondo.gaglio@unipa.it (R.G.)

Table S1. Taxa composition revealed by High-throughput sequencing analysis in Sicilian Canestrato Fresco cheeses.

Taxa	Samples					
	**SCF1**	**SCF2**	**SCF3**	**SCF4**	**SCF5**	**SCF6**
Actinobacteria	n.d.	n.d.	0.163	n.d.	n.d.	0.155
Bacteroidetes	1.268	n.d.	0.214	n.d.	n.d.	n.d.
Brochothrix	n.d.	n.d.	0.261	n.d.	n.d.	n.d.
Staphylococcus	n.d.	n.d.	n.d.	n.d.	n.d.	2.965
Lactobacillus	45.41	55.29	55.37	51.95	49.30	1.000
Lactococcus garvieae	2.136	n.d.	n.d.	n.d.	n.d.	n.d.
Other *Lactococcus ssp*	9.406	1.747	0.665	n.d.	3.977	9.786
Streptococcus	21.65	42.55	42.13	47.682	46.04	83.06
Other LAB[1]	n.d.	0.084	0.096	0.075	0.140	2.220
Aeromonadaceae	20.10	n.d.	n.d.	n.d.	n.d.	n.d.
Enterobacteriaceae	n.d.	n.d.	0.328	n.d.	0.112	n.d.
Pseudomonas	n.d.	n.d.	n.d.	0.155	n.d.	0.735

Results are expressed as relative abundances (%) of bacterial community. [1] Other LAB: reads defined only at order level as *Lactobacillales*. Abbreviations: SCF, Sicilian Canestrato Fresco cheese; 1–6, dairy factory; n.d., not detected.

Table S2. Volatile Organic Compounds determined by GC-MS in Sicilian Canestrato Fresco cheeses.

Chemical Compounds	Samples					
	SCF1	SCF2	SCF3	SCF4	SCF5	SCF6
Acids						
Acetic acid	16.96	14.53	13.38	16.54	13.78	14.91
Butanoic acid (Butyric acid)	28.19	30.47	32.09	27.51	29.88	31.99
Pentanoic acid (Valeric acid)	0.30	n.d.	0.23	0.19	0.32	0.22
Hexanoic acid (Caproic acid)	32.71	37.69	34.27	40.35	38.34	36.11
Octanoic acid (Caprylic acid)	6.00	4.84	3.24	5.76	5.66	4.35
Nonanoic acid (Pelargonic acid)	n.d.	1.00	1.18	0.44	0.38	1.15
Undecanoic acid	1.13	0.49	0.91	1.03	0.95	n.d.
Esters						
Ethyl butanoate (Ethyl butyrate)	2.17	0.89	1.84	0.51	1.01	1.21
Ethyl hexanoate (Ethyl caproate)	7.72	4.69	6.67	3.77	5.44	5.25
Ethyl nonanoate	0.53	0.53	0.58	n.d.	n.d.	0.53
Ethyl octanoate	0.25	0.44	0.68	1.42	0.56	0.12
Alcohols						
3-Methyl-1-butanol (Isoamyl alcohol)	1.03	1.16	1.09	0.80	0.92	1.29
3-Hexanol	0.92	1.09	0.47	n.d	1.05	0.08
2-Heptanol	0.54	n.d.	0.76	0.34	0.32	0.81
1-Octanol	0.16	0.23	n.d	0.12	0.2	0.32
Ketones						
2-Pentanone	0.10	0.13	0.06	0.14	0.1	0.08
2-Hexanone	0.52	0.26	0.57	0.15	n.d	0.44
2-Heptanone	n.d.	0.54	0.80	0.48	0.12	n.d.
2-Nonanone	0.84	0.81	n.d	0.33	0.24	0.84
Aldehydes						
benzaldehyde	0.12	0.11	0.17	0.07	0.09	0.14
Nonanal	0.07	n.d.	0.09	0.05	0.43	n.d.
2-Decenal	0.05	0.03	0.11	n.d	0.09	0.05
2-Octenal	n.d.	0.14	0.80	n.d.	0.12	0.10
∑Acids	85.29	89.02	85.3	91.82	89.31	88.73
∑Esters	10.67	6.55	9.77	5.70	7.01	7.11
∑Alcohols	2.65	2.48	2.32	1.26	2.49	2.50
∑Ketones	1.46	1.74	1.43	1.10	0.46	1.36
∑Aldehydes	0.24	0.28	1.17	0.12	0.73	0.29

Results are reported as percentage of triplicate determinations conducted at each sampling time point (peak area of each compound/total area of significant peaks) x100. Abbreviations: Abbreviations: SCF, Sicilian Canestrato Fresco cheese; 1–6, dairy factory; n.d., not detected.
